# Spotted Fever Group *Rickettsia* Infection and Transmission Dynamics in *Amblyomma maculatum*

**DOI:** 10.1128/IAI.00804-18

**Published:** 2019-03-25

**Authors:** Chanakan Suwanbongkot, Ingeborg M. Langohr, Emma K. Harris, Wellesley Dittmar, Rebecca C. Christofferson, Kevin R. Macaluso

**Affiliations:** a Vector-Borne Disease Laboratories, Department of Pathobiological Sciences, School of Veterinary Medicine, Louisiana State University, Baton Rouge, Louisiana, USA; Washington State University

**Keywords:** *Amblyomma maculatum*, *Rickettsia*, tick-borne pathogens

## Abstract

Tick vectors are capable of transmitting several rickettsial species to vertebrate hosts, resulting in various levels of disease. Studies have demonstrated the transmissibility of both rickettsial pathogens and novel *Rickettsia* species or strains with unknown pathogenicity to vertebrate hosts during tick blood meal acquisition; however, the quantitative nature of transmission remains unknown. We tested the hypothesis that if infection severity is a function of the rickettsial load delivered during tick transmission, then a more virulent spotted fever group (SFG) *Rickettsia* species is transmitted at higher levels during tick feeding. Using Amblyomma maculatum cohorts infected with Rickettsia parkeri or “*Candidatus* Rickettsia andeanae,” a quantitative PCR (qPCR) assay was employed to quantify rickettsiae in tick salivary glands and saliva, as well as in the vertebrate hosts at the tick attachment site over the duration of tick feeding. Significantly greater numbers of R. parkeri than of “*Ca*. Rickettsia andeanae” rickettsiae were present in tick saliva and salivary glands and in the vertebrate hosts at the feeding site during tick feeding. Microscopy demonstrated the presence of both rickettsial species in tick salivary glands, and immunohistochemical analysis of the attachment site identified localized R. parkeri, but not “*Ca*. Rickettsia andeanae,” in the vertebrate host. Lesions were also distinct and more severe in vertebrate hosts exposed to R. parkeri than in those exposed to “*Ca*. Rickettsia andeanae.” The specific factors that contribute to the generation of a sustained rickettsial infection and subsequent disease have yet to be elucidated, but the results of this study suggest that the rickettsial load in ticks and during transmission may be an important element.

## Abstract (precis)

Tick vectors are capable of transmitting several rickettsial species to vertebrate hosts, resulting in various levels of disease. Studies have demonstrated the transmissibility of both rickettsial pathogens and novel *Rickettsia* species or strains with unknown pathogenicity to vertebrate hosts during tick blood meal acquisition; however, the quantitative nature of transmission remains unknown.

## INTRODUCTION

Factors contributing to the rise in tick-borne rickettsial diseases (TBRDs) include increased numbers and ranges of the vectors and expanding identification of spotted fever group (SFG) *Rickettsia* species within tick populations. For example, Amblyomma maculatum, the Gulf Coast tick, was historically recognized only in the states lining the Gulf Coast and southern states along the Atlantic Ocean; however, recent surveys have detected established populations as far west as Arizona and as far north as Delaware (1–3). Coinciding with an expanding range, an increasing role in public health has also been realized for A. maculatum, which serves as the primary vector for an emerging rickettsial pathogen, Rickettsia parkeri, as well as other, less characterized rickettsial agents, including “*Candidatus* Rickettsia andeanae” (2, 4). Despite the recognition of multiple SFG *Rickettsia* species associated with a particular tick vector, the biological characteristics that govern rickettsial transmission and subsequent disease in vertebrate hosts are poorly defined.

Tick transmission of SFG *Rickettsia* species occurs both vertically and horizontally, and for most tick species, more than one rickettsial species is typically transmitted by either or both routes. The prevalence of *Rickettsia* species in tick populations is variable and may depend on the pathogenic nature, and subsequently the primary transmission route, of the bacterium (5). In A. maculatum, both single and dual infections with R. parkeri and “*Ca*. Rickettsia andeanae” have been reported (6–8). Recent field studies have identified “*Ca*. Rickettsia andeanae” in 9 to 62% of A. maculatum ticks collected from Kansas, Oklahoma, and Mississippi (7, 9). Similarly, the prevalence of R. parkeri infection in field-collected A. maculatum ticks ranges between 28 and 55% (7, 10, 11). Laboratory colonies of A. maculatum have been established with stable vertical transmission and demonstrated horizontal transmission of both “*Ca*. Rickettsia andeanae” and R. parkeri (12–15). As TBRDs increase in the United States (16), knowledge of the tick transmission potential and lesions associated with newly identified strains or species of SFG *Rickettsia* is required to help resolve the epidemiology of spotted fever rickettsioses.

The outcomes of vertebrate infection differ by rickettsial agent. Human infection with R. parkeri includes clinical symptoms such as fever, headache, diffuse myalgia, macular rash, and eschars associated with tick feeding sites (17). Extensive epidermal necrosis and superficial dermal necrosis, along with prominent lymphohistiocytic vasculitis of dermal vessels, are present (17–20). Although “*Ca*. Rickettsia andeanae” has not been associated with human disease (21), transmission of “*Ca*. Rickettsia andeanae” has been observed in C3H/HeJ mice and rhesus macaques exposed to “*Ca*. Rickettsia andeanae”-infected A. maculatum ticks in laboratory studies (12, 13). Immunohistochemistry (IHC) staining demonstrated the presence of rare coccobacilli in host skin at the tick attachment site. Compared with R. parkeri, “*Ca*. Rickettsia andeanae” induced less-severe dermatitis and only mild peripheral neutrophilia in the absence of changes in inflammatory cytokines and acute-phase proteins in the peripheral blood (12, 13). Although transmission of both agents has been demonstrated, the factors driving the distinct lesions are not known.

While biological and genetic variance in rickettsial strains/species is recognized, the factors driving lesion formation are still undefined (22–24). For other rickettsial pathogens, differences in bacterial strain/species loads within the host have been correlated with the severity of disease (25–27). The dynamics of “*Ca*. Rickettsia andeanae” infection in, and horizontal transmission kinetics by, A. maculatum have not been characterized. Therefore, the current study was designed to test the hypothesis that if infection severity is a function of the rickettsial load delivered during tick transmission, then a more-virulent SFG *Rickettsia* species is transmitted at greater levels during tick feeding. To examine this hypothesis, cohorts of constitutively infected A. maculatum ticks were used to characterize the rickettsial loads in their salivary glands and saliva, as well as in the skin of the vertebrate host at the tick attachment site over the course of tick feeding. Distinct differences between the rickettsial agents were identified, and the lesions associated with transmission were more severe in R. parkeri-exposed hosts. The data suggest that for the pathogenic SFG *Rickettsia* species R. parkeri, higher numbers of rickettsiae within the tick vector and tick saliva, and in subsequent transmission to the vertebrate host, contribute to infection outcomes.

## RESULTS

### R. parkeri loads were significantly higher than “*Ca*. Rickettsia andeanae” loads in both tick saliva and salivary glands.

Prior to the use of ticks in experiments, species-specific rickettsial infection was verified in all tick cohorts. Amplicons from portions of *ompA* were sequenced and were matched 100% to “*Ca*. Rickettsia andeanae” or R. parkeri *ompA* sequences deposited in NCBI databases (GenBank accession numbers KX158267.1 and KC003476.1).

Saliva was collected from ticks that had fed for 2, 6, or 10 days on a vertebrate host, and the presence of rickettsiae in the saliva was evaluated by quantitative PCR (qPCR) (Table 1). The prevalence of rickettsial DNA in saliva varied during tick feeding; 30% (6/20) to 40% (6/15) of saliva samples collected from “*Ca*. Rickettsia andeanae”-infected A. maculatum ticks contained rickettsiae at 2 and 6 days postattachment (dpa). The percentage of rickettsia-positive saliva samples increased significantly, to 100% (15/15), by 10 dpa. These data demonstrate that for “*Ca*. Rickettsia andeanae”-infected A. maculatum ticks, the presence of rickettsiae in the saliva of individual ticks was variable but increased with feeding activity. For R. parkeri-infected ticks, the percentage of *Rickettsia*-positive saliva samples (totaling 12, 16, or 12 at 2, 6, or 10 dpa, respectively) was consistently 100% at each time point assessed postattachment. Consequently, significantly more R. parkeri-infected ticks than “*Ca*. Rickettsia andeanae”-infected ticks secreted rickettsiae.

**TABLE 1:** Frequency of tick feeding, saliva collection, and detection of “*Ca*. Rickettsia andeanae” and R. parkeri in tick saliva during blood meal acquisition

Time (dpa)	Percentage (no. recovered/total no.) for ticks infected with:					
	“*Ca*. Rickettsia andeanae”			*R. parkeri*		
	Tick attachment	Saliva collection	qPCR positivity	Tick attachment	Saliva collection	qPCR positivity
2	100 (24/24)	83 (20/24)	30 (6/20)	100 (16/16)	75 (12/16)	100 (12/12)
6	100 (24/24)	62.5 (15/24)	40 (6/15)	100 (16/16)	100 (16/16)	100 (16/16)
10	100 (24/24)	62.5 (15/24)	100 (15/15)	100 (16/16)	75 (12/16)	100 (12/12)

**FIG 1:**
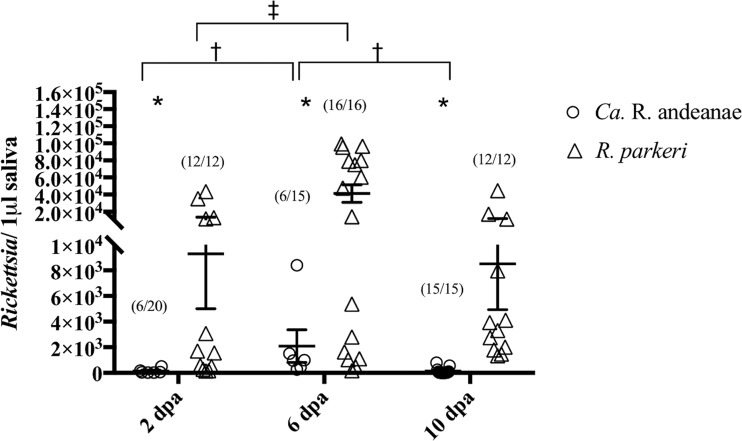
Quantity of rickettsiae in A. maculatum saliva as assessed by qPCR. The quantity of rickettsiae is expressed per microliter of tick saliva at 2, 6, and 10 days postattachment (dpa). Statistical analysis consisted of nonparametric tests, used to test for differences in the medians between and among groups, and a Kruskal-Wallis test followed by Dunn’s *post hoc* analysis for differences among days within each *Rickettsia* species, with a *P* value of ≤0.05. Wide horizontal bars represent the means; error bars represent the standard errors of the means. Symbols indicate significant differences between species (*) or between time points for “*Ca*. Rickettsia andeanae” (†) or R. parkeri (‡).

In *Rickettsia*-positive saliva samples, the total numbers of rickettsiae were enumerated via qPCR (Fig. 1). Within cohorts of infected ticks, the rickettsial loads in tick saliva varied significantly by day, with an ∼1.69-fold increase in “*Ca*. Rickettsia andeanae” loads detected between 2 and 6 dpa, followed by an ∼0.51-fold decrease between 6 and 10 dpa. A similar trend was identified in R. parkeri-infected samples, with a significant increase of ∼1.38-fold in rickettsial loads from 2 to 6 dpa. While R. parkeri loads decreased at 10 dpa, the difference from either 2 or 6 dpa was not significant. Comparison between tick cohorts identified significantly more rickettsiae in the saliva of R. parkeri-infected A. maculatum ticks than in that of “*Ca*. Rickettsia andeanae”-infected ticks (∼1.48, ∼1.48, and ∼2.88-fold at 2, 6, and 10 dpa, respectively). The data demonstrate that although both *Rickettsia* species showed 6-day peaks in the loads present in the saliva, the R. parkeri-infected cohort secreted significantly more rickettsiae in saliva over the course of blood meal acquisition.

**FIG 2:**
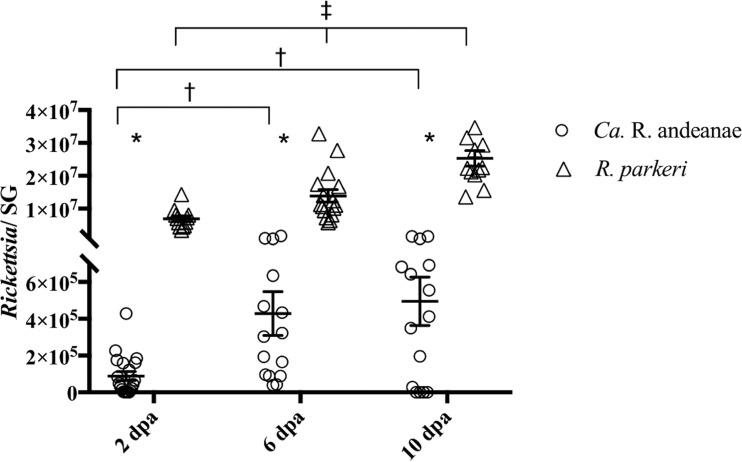
Quantity of *Rickettsia* species in A. maculatum salivary glands (SG) as assessed by qPCR. The quantity of rickettsiae is expressed per tick salivary gland at 2, 6, and 10 days postattachment (dpa). Statistical analysis consisted of nonparametric tests, used to test for differences in the medians between and among groups, and a Kruskal-Wallis test followed by Dunn’s *post hoc* analysis for differences among days within each *Rickettsia* species, with a *P* value of ≤0.05. Wide horizontal bars represent the means; error bars represent the standard errors of the means. Symbols indicate significant differences between species (*) or between time points for “*Ca*. Rickettsia andeanae” (†) or R. parkeri (‡).

**FIG 3:**
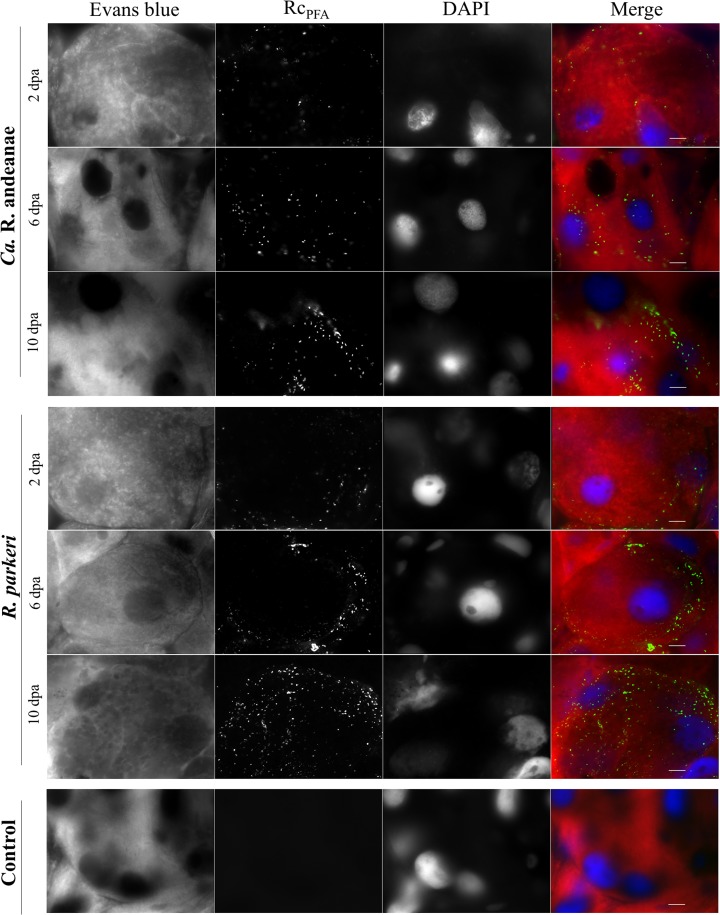
Immunofluorescence detection of rickettsiae in tick salivary glands. Positive staining for *Rickettsia* species (green) was identified in the salivary gland, which was counterstained with Evans blue and DAPI, showing as red and blue, respectively. Bars, 10 μm.

After saliva was collected, ticks were dissected, and salivary glands were recovered for the evaluation of rickettsial infection by qPCR and indirect immunofluorescence assay (IFA). Rickettsial loads increased in the salivary glands of both cohorts over the course of tick feeding (Fig. 2). There were significant increases in the total numbers of rickettsiae at 6 (∼1.17-fold) and 10 (∼1.20-fold) days of feeding over the number at 2 dpa in “*Ca*. Rickettsia andeanae”-infected A. maculatum ticks. Similarly, R. parkeri loads in salivary glands were significantly greater over time, with an ∼1.04-fold increase both from day 2 to day 6 of feeding and from day 6 to day 10 of feeding. When the cohorts were compared, R. parkeri-infected tick salivary glands had significantly greater rickettsial loads (∼1.46-, 1.29-, and 1.31-fold at 2, 6, and 10 dpa, respectively) than the salivary glands of the “*Ca*. Rickettsia andeanae”-infected cohort. Nonquantitative IFA analysis of salivary glands confirmed a higher density of staining in R. parkeri-infected samples than in “*Ca*. Rickettsia andeanae”-infected samples (Fig. 3). These data demonstrate that rickettsial loads increased in salivary glands during tick feeding, and they provide direct (qPCR) and indirect (IFA) evidence that R. parkeri was present in greater numbers than “*Ca*. Rickettsia andeanae.”

Further, there was a consistent difference in rickettsial loads between tick saliva and salivary glands at each time point assessed. Salivary glands tended to have higher numbers of rickettsiae than saliva. Interestingly, given the nature of the quantity of bacteria in the saliva over time, the relative proportion of rickettsiae in saliva to rickettsiae in salivary glands is correspondingly nonmonotonic. On day 6, for both species, the number of bacteria peaked in the salivary glands and was 54.5% and 62.6% of that found in individual salivary glands for “*Ca*. Rickettsia andeanae” or R. parkeri cohorts, respectively (Table 2).

**TABLE 2:** Temporal kinetics of “*Ca*. Rickettsia andeanae” and R. parkeri loads in saliva and salivary glands during blood meal acquisition

Time (dpa)	No. or proportion of rickettsiae in ticks infected with:					
	“*Ca*. Rickettsia andeanae”			*R. parkeri*		
	No. in saliva (log_10_)	No. in salivary gland (log_10_)	Relative proportion (saliva/salivary glands) (%)	No. in saliva (log_10_)	No. in salivary gland (log_10_)	Relative proportion (saliva/salivary glands) (%)
2	1.765	4.671	37.8	3.212	6.810	47.2
6	2.987	5.483	54.5	4.416	7.049	62.6
10	1.534	5.615	27.3	3.558	7.350	48.4

### R. parkeri is transmitted to vertebrate host skin in greater numbers, and causes more-severe lesions, than “*Ca*. Rickettsia andeanae.”

**FIG 4:**
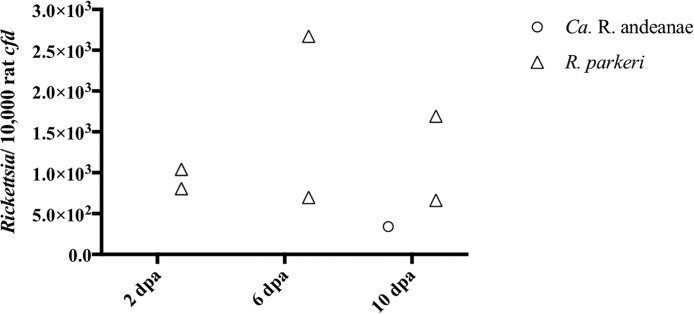
Quantity of rickettsiae in host skin at the tick attachment site as assessed by qPCR. The number of rickettsiae is expressed per 10,000 copies of the rat *cfd* gene at 2, 6, and 10 days postattachment (dpa).

Vertebrate host skin at tick attachment sites and in areas away from the attachment sites were recovered and were assessed for rickettsial DNA and lesions. DNA from “*Ca*. Rickettsia andeanae” was detected in the skin at the tick infestation site only at 10 days of tick feeding. Significantly more R. parkeri DNA than “*Ca*. Rickettsia andeanae” DNA was detected in skin samples at 2, 6, and 10 days of tick feeding (Fig. 4). Additionally, the highest numbers of rickettsiae were detected in the skin of hosts at 6 days of feeding in the R. parkeri-infected cohort. No rickettsial DNA was detected in blood or in skin distant from the tick attachment site at any time point.

**FIG 5:**
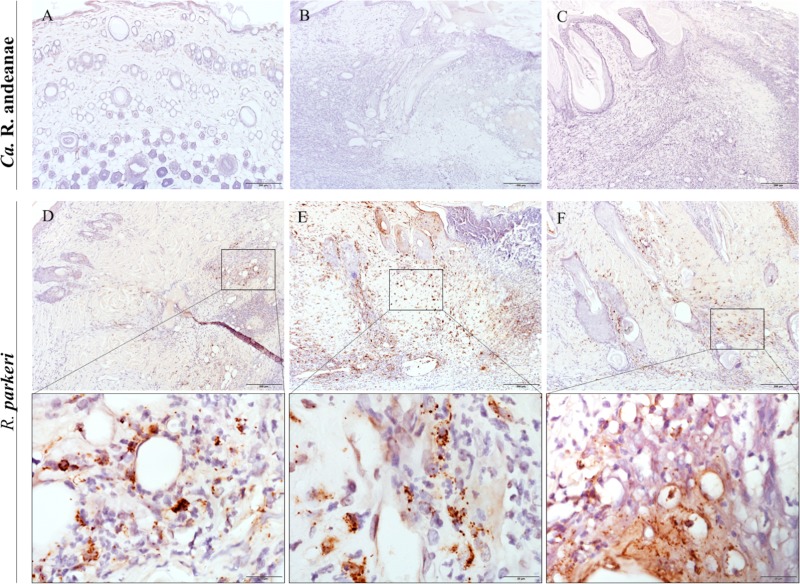
(A through C) Anti-*Rickettsia* immunohistochemistry demonstrated the absence of “*Ca*. Rickettsia andeanae” at the tick feeding site 2 (A), 6 (B), and 10 (C) days postattachment. (D through F) Conversely, variable numbers of organisms were observed in the skin of rats at the feeding sites of R. parkeri-infected ticks. Inflammatory cells containing positive, brown-staining coccobacillary rickettsial organisms were present in one aggregate or scattered aggregates at 2 (D) and 10 (F) dpa. Inflammatory cells with positive, brown-staining organisms were widespread throughout the skin at 6 dpa (E). Bars, 200 μm (magnification, ×10) for panels and 20 μm (magnification, ×100) for insets.

**FIG 6:**
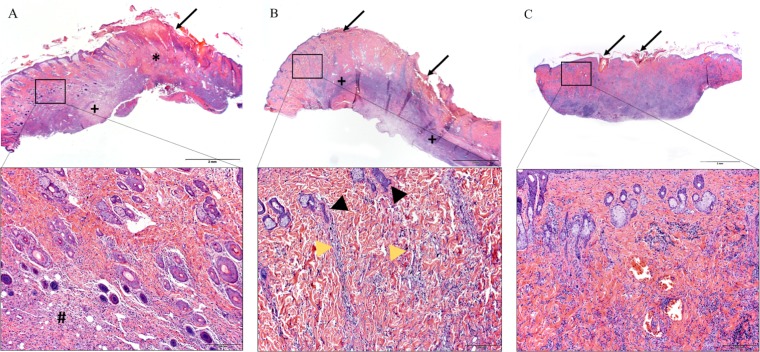
(A and B) Histopathology of rat skin at the feeding site of *Rickettsia*-infected ticks at 6 dpa. (A) “*Ca*. Rickettsia andeanae.” The skin had severe focal epidermal necrosis and dermal inflammation with a large area of fibrin exudation (*) and extensive subcutaneous granulating fibrosis (+). (Inset) There were multiple normal-appearing adnexa throughout the dermis, with a mild mixed inflammatory infiltrate (#) at the deep aspect. (B) In R. parkeri-exposed hosts, there was extensive epidermal necrosis with a band of fibrosis and inflammation replacing the panniculus carnosus (+). (Inset) Some inflammatory cells tracked up from the inflamed and fibrotic subcutis along the deep and mid-derma vascular plexi (yellow arrowheads). Many adnexa in the dermis were atrophied or “faded” (black arrowheads). (C) Uninfected-tick feeding site at 10 dpa, with mild epidermal necrosis, restricted to the tick attachment site, accompanied by deep dermatitis and panniculitis. The arrows indicate tick attachment sites. Bars, 2 mm (magnification, ×1.5) for panels and 200 μm (magnification, ×40) for insets.

Anti-*Rickettsia* immunohistochemical staining of the skin sections collected at the tick feeding sites revealed variable numbers of positive coccobacilli in the inflamed dermides of the rats of the R. parkeri-infected groups only. Abundant positive staining was noted at 6 dpa, and less at 2 and 10 dpa (Fig. 5D to F). Positively immunostained coccobacilli were mostly within macrophages; fewer were within neutrophils. No immunostaining was observed at any time point in the rats of the “*Ca*. Rickettsia andeanae”-infected group (Fig. 5A to C). Among the rats exposed to “*Ca*. Rickettsia andeanae”-infected ticks, one of three had mild dermal inflammation at 2 dpa. At 6 dpa, there was much more extensive dermatitis and panniculitis, with partial tissue effacement by large regions of fibrinosuppurative exudate encircled by granulating fibrosis. Necrotic tissue extruded over the skin surface at the tick attachment sites, with heavy secondary bacterial colonization (Fig. 6A). Cutaneous necrosis and accompanying inflammation were even more extensive at 10 dpa, accompanied by abundant granulation tissue in the deep dermis and subcutis (Table 3). Moderate numbers of large (reactive) lymphocytes infiltrated the granulation tissue, often in the form of dense aggregates within lymphatics. The histologic alterations in rats exposed to R. parkeri-infected ticks were similar overall to those in rats exposed to “*Ca*. Rickettsia andeanae”-infected ticks, with necrosis and fibrinosuppurative exudate encircled by granulating fibrosis already apparent at 2 dpa and still present as chronic active inflammatory lesions at 6 dpa (Fig. 6B) and 10 dpa (Table 3). Nonetheless, in contrast to the “*Ca*. Rickettsia andeanae”-infected cohort, where the alterations were extensive in both the deep dermis and the subcutis, the lesions in the R. parkeri-infected rats predominated in the subcutis at all time points examined, with inflammation in the overlying dermis centered mostly on the deep and mid-dermal vascular plexi. These alterations were typically accompanied by stromal pallor in the deep dermis, atrophied or “faded” hair follicles, and, in some animals, superficial coagulative necrosis, consistent with ischemic change. This lesion pattern was unique and distinguished this group from the “*Ca*. Rickettsia andeanae”-infected rats. Overt vasculitis was variably apparent in all rats, characterized by intramural and perivascular fibrin deposition, endothelial cell degeneration or necrosis, and/or intramural inflammatory cell infiltration (Table 3). Occasional intralesional thrombosed vessels were also apparent. While vascular compromise was suspected to be more severe in the R. parkeri-infected rats due to evidence of ischemic change in the dermis, this was in fact not always apparent, likely because the vascular alterations were masked by the extensive inflammation and fibrosis at the deep aspect of the skin sections.

**TABLE 3:** Cutaneous alterations in response to “*Ca*. Rickettsia andeanae” and R. parkeri at the tick infestation site

Animal	Score^*a*^ at the indicated time (dpa)											
	Epidermal necrosis			Dermatitis			Panniculitis			Anti-*Rickettsia* IHC		
	2	6	10	2	6	10	2	6	10	2	6	10
“*Ca*. Rickettsia andeanae” 1	0	++	+++	0	++	+++	0	+++*	+++*	0	0	0
“*Ca*. Rickettsia andeanae” 2	**+**	++	+	0	++	+++	0	+++*	+++*	0	0	0
“*Ca*. Rickettsia andeanae” 3	0	++	0	++	++	++	+	+++	+++	0	0	0
R. parkeri 1	0	++	++	++	++	++	+++*	+++*	+++*	++	++	++
R. parkeri 2	+	++	+++	++	++	++	+++*	+++*	+++*	++	+++	+
Uninfected tick	ND	ND	+	ND	ND	++	ND	ND	+++	ND	ND	ND

a Histopathological findings associated with *Rickettsia*-infected tick feeding in rats. Scores are as follows: 0, absence of the specified parameter; +, mild histologic change (finding is rare to infrequent at high power); ++, moderate histologic change (change is found in multiple high-power fields, or large foci are present in selected areas); +++, marked histologic change (changes are frequently observed in multiple high-power fields, or change is severe in focal areas); ND, not determined. Asterisks indicate diffuse inflammation, as opposed to perivascular inflammation (indicated by the lack of an asterisk). Each score at each time point represents findings for one individual rat.

## DISCUSSION

In the current study, the rickettsial infection dynamics of R. parkeri and “*Ca*. Rickettsia andeanae” within the tick saliva and salivary glands, and at the tick-host interface, were compared. Using qPCR and microscopy, distinct profiles for each agent associated with rickettsial load and *Rickettsia*-induced lesions were identified. In early tick feeding, the prevalence of R. parkeri was greater than that of “*Ca*. Rickettsia andeanae” in saliva samples, although all tick salivary glands were infected with the respective agents. The prevalence of “*Ca*. Rickettsia andeanae” in tick saliva reached 100% only after 10 days of feeding, suggesting that the variability in *Rickettsia*-positive saliva within the “*Ca*. Rickettsia andeanae” cohort may be associated with tick attachment time. Since low levels of “*Ca*. Rickettsia andeanae” have been detected in tick feeding lesions of vertebrate hosts after 4 days of tick attachment (12), the factors contributing to a feeding threshold prior to the secretion of rickettsiae into the saliva and their transmission to the host need to be identified.

Quantitative analyses of “*Ca*. Rickettsia andeanae” in tick saliva and salivary glands showed lower rickettsial loads in both samples than in those from the R. parkeri-infected cohort of ticks. A large variability in rickettsial load has been identified in unfed ticks (28), and unique infection levels have been identified for *Rickettsia* species within the same tick vector species (29). Likewise, quantification of *Rickettsia* species has been described in whole ticks and, at the organ-specific level, during tick feeding. For example, loads of *Rickettsia* sp. phylotype G021 increased 57.5-fold in fed Ixodes pacificus ticks over those in unfed ticks (30). A significant increase (∼38.4-fold) in the load of a recently described rickettsial pathogen, Rickettsia massiliae, was observed in the salivary glands of Rhipicephalus sanguineus ticks after 6 days of feeding (31). Though at different magnitudes, both R. parkeri and “*Ca*. Rickettsia andeanae” loads increased in salivary glands over the course of feeding. Interestingly, for both agents, there was a peak in the mean number of rickettsiae present in the saliva at 6 dpa. While more R. parkeri organisms were present in the salivary glands and saliva of infected ticks than “*Ca*. Rickettsia andeanae” organisms in the corresponding infected cohort, the level of rickettsiae in saliva as a percentage of rickettsiae in the salivary glands did not differ between the cohorts. Rickettsial loads in ticks will increase as ticks acquire a blood meal (32), plausibly through metabolic coupling between rickettsiae and ticks (33). Previously, when the ratio of the rickettsial load to the number of tick host cells was assessed, Rickettsia amblyommatis levels remained relatively constant over the course of feeding in the tick vector Amblyomma americanum (34). When the current study compared two different *Rickettsia* species and enumerated rickettsiae in tick salivary glands and saliva during feeding, distinct rickettsial loads were identified, suggesting that greater *Rickettsia* virulence resulted in increased rickettsial loads and transmission efficiency.

Distinct transmission phenotypes were observed in vertebrate hosts exposed to R. parkeri-infected ticks and those exposed to “*Ca*. Rickettsia andeanae”-infected ticks. Rickettsiae were detected in the host skin by both qPCR and microscopy at all time points assessed for the R. parkeri-exposed group, while “*Ca*. Rickettsia andeanae” was detected in host skin only at 10 dpa. Case studies suggest that the minimal feeding time required for naturally infected ticks to transmit R. parkeri is approximately 8 h (35, 36), supporting the observation of transmission at 2 days post-tick attachment in the current study. Tick transmission of “*Ca*. Rickettsia andeanae” to vertebrate hosts during tick feeding has only recently been recognized, with rickettsiae being detected by qPCR or by microscopy in as few as 4 days post-tick exposure (12, 13). Disparities in detection can be due to low levels of rickettsiae in the skin or to sampling techniques that may prevent identification by either qPCR or IHC. Cutaneous alterations at the tick infestation site varied in animals exposed to either cohort of infected ticks. Dermatitis and panniculitis was more frequently observed in the skin at 2 dpa in the R. parkeri-exposed groups. Additionally, extensive epidermal necrosis was observed at the tick attachment site for the R. parkeri-exposed group at 6 dpa. The severity of the lesions coincided with the molecular detection of rickettsiae at the feeding site. Nonetheless, while the lesions in R. parkeri-infected rats were distinctly more severe in that they had evidence of ischemic change in the dermis, presumed to be due to greater vascular compromise than that for “*Ca*. Rickettsia andeanae”-infected rats, gross analysis of the lesions would not suffice to differentiate the infections. Microscopic analysis, paired with IHC and PCR assays, would be required to accurately characterize the infections.

In the United States, an increase in tick-borne spotted fever rickettsiosis has occurred over the past 12 years, with human infections attributed to Rickettsia rickettsii, R. parkeri, and *Rickettsia* sp. strain 364D (16). Coinciding with increased incidence are reports of other SFG *Rickettsia* infections associated with tick feeding, and several studies have reported tick transmission of what are considered rickettsial symbionts to vertebrate hosts. For example, both Rickettsia montanensis and R. amblyommatis have been implicated in tick-derived human infections that result in the formation of a rash (37, 38). In addition, in two laboratory models, A. maculatum transmitted “*Ca*. Rickettsia andeanae” to both mice and rhesus macaques during tick feeding (12, 13), findings similar to those with the rat model employed in the current study. Several factors may contribute to transmission, including rickettsial virulence and/or strain variation, which is common among *Rickettsia* species. Genetic differences have been identified among different strains of R. rickettsii (22, 39), and a less virulent strain, R. rickettsii strain Iowa, was able to replicate in the guinea pig model in the absence of apparent disease (40). Likewise, strain variability in R. amblyommatis may account for differences in the lesions observed in a guinea pig model of infection (23, 24). Many laboratory models have examined disease in the absence of the tick vector, specifically the contribution of saliva to rickettsial infection. However, it is evident that tick saliva influences the host response, possibly facilitates rickettsial dissemination, and alters the lesions in the vertebrate host (12, 13). Few studies have explored the strain variability of A. maculatum (41, 42), and none have demonstrated tick strain-dependent differences in transmission efficiency for any known rickettsial pathogen. However, it is possible that tick strain differences contribute to the transmission efficiency of rickettsial pathogens and nonpathogens alike. To determine if tick strains influence rickettsial transmission, future studies will require characterized rickettsial isolates and a suitable infection/acquisition bioassay to control for tick variables. The current study identifies a tick-derived infectious dose and distinct lesion pattern in hosts exposed to R. parkeri-infected ticks and suggests that the rickettsial loads in the tick salivary glands and saliva, as a function of the pathogenic nature of R. parkeri, may influence the lesion pattern. Indeed, bacterial loads for a “minimal infectious dose” have been recognized and have been associated with infection outcomes for closely related rickettsial organisms, including Anaplasma marginale and Orientia tsutsugamushi (25–27). Thus, in the absence of observable lesions associated with “*Ca*. Rickettsia andeanae” infection via ticks, it is plausible that the agent is not delivered in a dose sufficient to consistently induce disease. Further studies are required to identify potentially synergistic interactions between vector saliva and *Rickettsia* species so as to better understand the transmission kinetics that result in disease.

## MATERIALS AND METHODS

### Tick cohorts.

Two separate colonies of A. maculatum were maintained at the Louisiana State University School of Veterinary Medicine (LSU-SVM) as described previously (12, 13, 43). All animals were used in the experiments with permission from the Institutional Animal Care and Use Committee at LSU-SVM (protocol 15-115). The Sand Hill strain of R. parkeri-infected A. maculatum was established at the University of Southern Mississippi (14). The “*Ca*. R. andeanae”-infected A. maculatum cohort was derived from a colony established at LSU-SVM as described by Grasperge et al. (13). Prior to experiments with adult ticks, *Rickettsia*-infected nymphs (*n* = 10) were screened for *Rickettsia* via traditional PCR using species-specific primers as described by Jiang et al. (44) (Table 4). To confirm rickettsial species, a partial sequence for the gene encoding the outer membrane protein (*ompA*) was amplified from nymphal genomic DNA (gDNA) samples using primers 190.70 (45) and 190.701 (46) (Table 4). Amplicons (∼640 bp) were sequenced and nucleotides compared to those in the GenBank database (NCBI) using the BLAST function.

**TABLE 4:** Primers and probes used for detection of the vector, *Rickettsia* species, and host

Primer set or probe	Sequence^*a*^ (5′–3′)	Partial gene amplified	Reference or source
Rr190.70	ATGGCGAATAATTCTCCAAAA	*Rickettsia* sp. *ompA*	45
Rr190.701	GTTCCGTTAATGGCATCT		46
R.and957F	CGCTGGACAAGTTTATGCTCAAG	“*Ca*. Rickettsia andeanae” *ompB*	44
R.and1062R	GGCAGTAGTACCGTCTGTACCAC		
R.and1003	FAM–CGCGATGAGGCGGACAGGTAACTTTTGATCGCG–BHQ-1		
RpompB129F	CAAATGTTGCAGTTCCTCTAAATG	R. parkeri *ompB*	44
RpompB224R	AAAACAAACCGTTAAAACTACCG		
RpompB	FAM–TTTG+A+G+C+A+G+AC–IABKFQ^*b*^		12
A.macMIF.18F	CCAGGGCCTTCTCGATGT	A. maculatum *MIF*	7
A.macMIF.99R	CCATGCGCAATTGCAAACC		
A.macMIF.63	HEX–TGTTCTCCTTTGGACTCAGGCAGC–BHQ-1		
Ratcfd121F	GCTTCAGTGCAAGTGAATGG	Rat *cfd*	50
RatcfdRev	TGCCACTCACACTCCATCC		This paper
RatcfdHex	HEX–TGGATGAGCAGTGGGTGCTGA–BHQ-1		This paper

a FAM, 6-carboxyfluorescein; BHQ-1, black hole quencher; IABKFQ, Iowa Black dark quencher; HEX, hexachlorofluorescein.

b A plus sign denotes the use of a locked nucleic acid.

### Tick feeding and sample collection.

At a ratio of 2:1 (female to male), “*Ca*. Rickettsia andeanae”- or *R. parkeri*-infected adult *A. maculatum* ticks were encapsulated on Sprague Dawley rats (≥5 weeks old) and allowed to feed for 2, 6, or 10 days postattachment (dpa), at which time female ticks were forcibly removed and hosts were euthanized for tissue collection. Eight female ticks were recovered from each host for each time point assessed. Feeding assays were carried out in duplicate for the R. parkeri-infected ticks and in triplicate for the “*Ca*. Rickettsia andeanae”-infected cohort. For saliva collection, individual ticks were taped to a glass microscope slide, and a prepulled 25-μl microcapillary pipette (Kimble Chase Life Science) was applied over the hypostome. Salivation was then induced by applying 5 μl of 3% pilocarpine HCl (MP Biomedicals) in methanol to the dorsum three times over the course of 4 h while ticks were kept in a 37°C incubator (47). Saliva from individual ticks was stored at –20°C prior to gDNA extraction. After saliva collection, individual ticks were surface sterilized via serial washes for 5 min in 70% ethanol and 1% bleach, followed by three rinses with sterile distilled water. Pairs of salivary glands were dissected from individual ticks; one gland was stored at –20°C for gDNA extraction and the other fixed in 4% paraformaldehyde for 15 min on a multiwell slide for immunofluorescence assay. For vertebrate hosts, whole blood was collected via cardiocentesis and was frozen at −20°C. Samples of rat skin at the tick attachment site and at a distal location free of parasitism were collected from each group. Tissues were halved and were either stored at −20°C for DNA extraction or placed in 10% neutral buffered formalin for histopathology.

### DNA extraction and qPCR.

The DNeasy Blood and Tissue kit (Qiagen) was used for extraction of gDNA from tick saliva and salivary glands, rat skin representing attachment or distal sites, and blood samples according to the manufacturer’s instructions. Briefly, individual tick salivary glands were snap-frozen in liquid nitrogen and ground with a pestle, and skin samples in lysis buffer were transferred to Eppendorf Safe-Lock microcentrifuge tubes (Eppendorf) containing 2 sterile 3-mm stainless steel beads (Qiagen) to be disrupted via TissueLyser (Qiagen) for 2 cycles of 30 s at 30 Hz. Proteinase K was then added and samples incubated at 56°C for ∼16 h prior to gDNA extraction. An environmental DNA extraction control was included with the experimental samples.

For the detection of DNA via qPCR, species-specific primers and fluorescently labeled probes for “*Ca*. Rickettsia andeanae” *ompB* and R. parkeri *ompB* and for host genes (A. maculatum *MIF* and rat *cfd*) were used. Rat *cfd* primers were modified for this experiment by designing a new reverse primer and a new probe (Table 4). For qPCR, the following were included for all runs: standard dilutions, experimental samples, environmental extraction controls, and no-template controls. All qPCRs were performed using iTaq Universal Probe supermix (Bio-Rad) and a LightCycler 480 II system (Roche), as described previously by Thepparit et al. (48), with a modified preincubation step of 95°C for 3 min. Amplicons for each set of primers were incorporated into pCR4-TOPO and the resulting plasmids diluted to serve as internal standards for all experimental samples. Genomic copy numbers of rickettsiae were calculated for tick samples, and the presence of tick DNA in the salivary glands was confirmed with the *MIF* reaction. In vertebrate hosts, rickettsial infection density was calculated as the ratio of the rickettsial copy number to the rat cell copy number.

### Immunofluorescence assay.

Rickettsiae were visualized in salivary glands as described by Harris et al. (49). Briefly, fixed tick salivary glands were first permeabilized with 0.1% Triton X-100 in phosphate-buffered saline (PBS) for 15 min and then blocked with 3% bovine serum albumin (Sigma-Aldrich) in PBS for 1 h. Slides were then washed three times with 0.01% Triton X-100 in PBS and were incubated with the diluted polyclonal antibody RC_PFA_ (1:200) for 1 h, followed by incubation with an Alexa Fluor 488-conjugated goat anti-rabbit antibody (1:1,000; Molecular Probes). Salivary glands were counterstained with 0.1% Evans blue (Sigma) in PBS for 30 min. Coverslips were mounted with Vectashield HardSet antifade mounting medium with DAPI (4',6-diamidino-2-phenylindole) (Vector Laboratories Inc.) for nuclear staining. Samples were visualized using an Observer Z1 microscope (Zeiss).

### Histopathology and IHC.

Host skin tissue samples were fixed overnight in 10% neutral buffered formalin. Tissues were routinely embedded in paraffin, and 4-μm-thick sections were cut for hematoxylin and eosin (H&E) staining and immunohistochemistry (IHC). Rickettsial organisms were visualized using an indirect immunoalkaline phosphatase technique with a 1:2,000 dilution of the polyclonal antibody RC_PFA_ as described by Banajee et al. (12). A board-certificated veterinary anatomic pathologist examined the sections in a blinded manner.

### Statistical analysis.

Daily infection rates of ticks were tested using a z-test to determine differences relative to rickettsial species. Since the data were nonnormal (*P*, >0.05 by the Shapiro-Wilk test), a Kruskal-Wallis test was used to test for differences in log_10_-transformed rickettsial quantities in positive ticks within and between species in both saliva and salivary glands, and Dunn’s *post hoc* test was used to identify the time points at which differences were observed. All statistical analyses were performed using R (version 3.4.3) in R Studio (version 1.1.383), and significance was assessed at the 95% confidence level. Observed differences in the skin are reported, but sample sizes were insufficient for statistical analyses. Zero values could not be transformed and were kept as zeroes in the data.
